# Combining Microfinance and Health in Reducing Poverty-Driven Healthcare Costs: Evidence From the Philippines

**DOI:** 10.3389/fpubh.2020.583455

**Published:** 2020-10-08

**Authors:** Lolita L. Aranas, Rasheda Khanam, Mohammad Mafizur Rahman, Son Nghiem

**Affiliations:** ^1^Graduate Research Studies, University of Southern Queensland, Toowoomba, QLD, Australia; ^2^Graduate Research Studies, Jose Rizal University, National Capital Region, Mandaluyong, Philippines; ^3^School of Commerce, Center for Health Research, University of Southern Queensland, Toowoomba, QLD, Australia; ^4^Centre for Applied Health Economics, Griffith University, Brisbane, QLD, Australia

**Keywords:** microfinance, healthcare costs, medical care costs, poverty, Philippines, cooperative

## Abstract

The role of microfinance in alleviating poverty and poor health is significant. Its health programs have been shown to improve healthcare utilization and strengthen a healthcare system. In the Philippines, microfinance's widespread presence is seen as instrumental in achieving the objectives of *Healthy Philippines 2022*, particularly in reducing poverty-driven healthcare costs. However, little is known on how microfinance can reduce the cost of healthcare services and treatment. Also, few studies that consider the practice of integrated microfinance and health programs in the Philippines have been seen. Secondary data was used to explore the structure and function of microfinance and health initiatives and their influence in mitigating healthcare costs. A review criterion was developed to examine the data using the three key elements identified in Ruducha and Jadhav's framework: organisational arrangement, health products and health outcomes. Findings revealed that most health initiatives are delivered through partnerships and collaboration, could favour a reduction in healthcare costs and protection from out-of-pocket health expenditure. They are designed to operate in three structures—subsidised or outreach, microinsurance and health loans, and patronage refunds. The cooperative's business venture providing pharmaceuticals facilitated access to affordable medicine and offered its members financial viability. Health loans and microinsurance also offered healthcare cost reductions; however, uptakes are low. The study found no data to assess the output of the completed health initiatives. More studies that will evaluate the integrated MFI health initiatives are recommended to further identify gaps, outcomes, or impacts of the program.

## Introduction

This study is guided by the idea that within the context of microcredit, financial services are not only confined within the premises of business investment but also investments in health (1). The vast number of microfinance institutions (MFI) can be instrumental in supporting a healthcare agenda (2)—one of which is to reduce poverty-driven healthcare costs (3). This study explores the integrated microfinance-health programs and their contributions to healthcare in the Philippines.

Situated in Southeast Asia, the Philippines has an estimated population of 109 M in 2019 (4) and considered one of Asia's fastest-growing economies (5). But despite an optimistic financial picture, poverty remains. With one-fifth of its population living in poverty (6), the burden of illness and its consequences continue to challenge the healthcare system.

In 2018, Universal Healthcare (UHC) Bill enrolled Filipino citizens in the National Health Insurance Program (PhilHealth) and prescribed reforms in the health system. The “no-balance billing” policy which means that “no other fees or expenses will be charged or be paid for by the indigent patients above and beyond the packaged rate during their confinement period” (7) was introduced. The government increased its budget allocation and safety nets for health (8) to help achieve the goals of health agenda *Healthy Philippines 2022*, which advocates the UHC call for improving the three coverage dimensions—population coverage, quality of services and cost of services (9). Despite reforms, financial health protection remains limited. Out-of-pocket spending continues to dominate as source for health care financing (10), while others forgo medication. ~1.5 M Filipinos are pushed to poverty each year due to healthcare expenditure (3).

To curb poverty and poor health, the government vigorously pushed sectoral collaboration and partnerships—a strategy which is seen to improve service delivery with stakeholder support (11, 12). Leatherman and Dunford (2) reported that one of the potential community linkages to health is microfinance, which is one of the country's poverty reduction and development initiatives. In 2018, about 10.7 M Filipinos registered for memberships to 18,065 cooperatives. With its expansive membership, health advocates can use cooperatives to reduce poverty by integrating health into the microcredit system (2).

Studies showed that microfinance helps to improve population health (13). MFIs contributed to improving healthcare utilisation (14, 15), mitigating health shocks (16), and strengthening healthcare systems (17). However, little is known about how microfinance can reduce the cost of healthcare services. Also, there are few Philippine studies into the practice of integrated microfinance and health programs.

This study evidences the practice of integrated microfinance and health programs in the Philippines. It reviews the design and implementation of health initiatives of Barbaza Multipurpose Cooperative (BMPC), an MFI in the Philippines. The BMPC provides microfinance with health and developmental services in Western Visayas region whose population is ~7.8 M, with a poverty incidence of 16.4%, and 4.2% of the population is considered extremely poor (6).

This study utilised two complementary models—one which identifies the realm that microfinance needs to address in facilitating healthcare access, and the other illustrates the structural and functional design of an MFI-health program. Leatherman and Dunford (18) concluded that inadequate health information, insufficient accessible, affordable and effective healthcare services, and inadequate financing for health are priority issues for health program pathways. Meanwhile, Ruducha and Jadhav's (19) framework maps the relationship between institutional arrangements, health services, products and outcomes which could improve the health of microfinance members.

## Method

This study utilised secondary data. A review criterion using the three key elements in Ruducha and Jadhav's (19) framework—organisational arrangement, health products and health outcomes. The review criteria expanded upon the questions:

What is the structure of the health initiative?
How does the health initiative function?
Is the health initiative in line with national/local needs and priorities?
What health products are offered?
Does the health offering facilitate in reducing the cost of healthcare service?
Have short-term results been achieved or be likely to be met?

### Data and Findings

BMPC had an estimated 85,000 members in 2018, mostly women. Members are categorised into (1) regular or associate and (2) participation standing, also called segmentation criteria. Regular members are those with minimum required shared capital or more, have the right to vote; while associate members have below minimum shared capital and no voting rights. About 71% of the total BPMC members are registered into regular membership.

The second category, segmentation criteria, groups members into Diamond, Gold, Silver, Bronze, and Brass. Segmentation criteria are based on years of membership, amount of share capital and savings, loan standing, and attendance at cooperative assemblies. To aid in the description and data analysis, this study has two categories—Category A (Diamond, Gold, and Silver) and Category B (Bronze and Brass).

The segmentation criteria define the members' healthcare incentives. Category A members receive up to US$12 daily hospitalisation benefit for a 3–5 days confinement per year; however, none for Category B. Category A comprises a minute portion (2.27%) of the total segmentation criteria membership. The majority (97.73%) of the BMPC members belong to Category B. The ratio between categories indicates that most of the members do not receive the medical care cost-reducing incentive.

BMPC allocates medical funds for its staff medical benefits and other health services. Since 2014, the medical fund allocation increased from US$12,000 to about US$25,000 in 2018. Medical benefits include microhealth insurance premiums, primary care, annual check-ups, and drug testing. Community health services also share a part of the medical fund. BMPC and its partners cater to the following health initiatives:

#### Medical Mission

Medical missions are subsidised activities in which local medical professionals participate to provide primary health care, including free prescription eyeglasses to identified beneficiaries. Blood monitoring is offered by trained personnel; however, no data is available to evaluate the service utilisation of its outcome. In the 2019 annual plan, branch clinic set-ups manned by adjunct health professionals were considered a priority.

#### Feeding Program

Under the umbrella “Adopt a School Program,” a BMPC feeding program collaborated with the local primary public school and spent about US$4,500 on these initiatives in 2018. No available data details the activities completed.

#### Micro-Health Insurance

BMPC partnered with a Health Maintenance Cooperative (HMC), 1CoopHealth, which offers health package to low income and informal sectors. With an annual premium of US$75, members are covered with US$1,200 per illness for in-patient treatment, US$600 for emergency treatment, death benefits of up to US$400, and preventive health care. BMPC covers the micro-health insurance enrolment and annual premium of all its staff; while other members enrol and pay the premiums voluntarily. Despite encouragement, the popularity of the health scheme has been consistently low. In 2019, 370 out of 84,430 members were enrolled. No data to back-up an analysis of this downtrend is available. The 2019 strategic plan indicated using intensive information dissemination on 1CoopHealth to improve the number enrolled.

#### Medical Loan

Another health initiative provided, especially during medical emergencies, is an alternative health financing scheme in the form of a medical loan. There was an increasing number of borrowers between 2015 and 2018, prompting the BMPC to increase its medical funds. But overall, only 6.1% members took up the medical loan during that period. With the shallow popularity of 1CoopHealth, it can be inferred that medical loans were preferred for medical emergencies. No data, however, was available to identify which medical emergencies used the medical loans and why it was preferred over health insurance.

#### I-Coop Program

This program assists members to register into PhilHealth and subsidises the premiums of 50 selected beneficiaries. PhilHealth registration is compulsory coverage for all Filipinos, and its execution is built on existing community initiatives (7). As the country's national insurance, it covers services that focus on in-patient care, with selected outpatient care available.

#### Blood Bank Coop and Anti-dengue Project

Haemorrhagic dengue fever cases have spiked in Western Visayas since 2015 (6), which lead to increased demand for whole blood and blood components. In response, BMPC corroborated with PNRC to establish the Cooperative Blood Bank. Periodic blood donation is conducted in all of its nine branches to sustain supply in the Cooperative Blood Bank. Blood donors are cooperative members themselves. No data was made available to determine the amount of blood and its components utilised by its members.

Additionally, BMPC partnered with local hospitals to provide free dengue test kits, intravenous fluids, oral rehydration fluids, antipyretics and vitamins, etc.

#### Cooperative Pharmacy

The cooperative pharmacy allows members access to affordable pharmaceutical products at discounted prices. Under Generic Law, medicines are subject to approved maximum retail price and affordable (20). Additionally, the cooperative members receive patronage refunds for every purchase made. Since its opening in 2017, the sales volume rapidly increased, showing a surge of demand for pharmaceutical products among members.

## Discussion

The significant increase in BMPC members in the past 5 years may suggest efficient delivery of quality services to its members. BMPC branches are located strategically close to their members. Consistent interaction with members helps cooperatives to design mechanisms appropriate to their members (21), thus could contribute to improving the health of BMPC members. An indicator of cooperative service competency is the increase in regular members compared to associated members. Regular membership can be attributed to the members' commitment to the cooperative. What delineates a regular and associate member is the right to vote. This qualifying criterion stipulates the cooperative's commitment to promoting best practices and maintaining a transparent and democratic microcredit structure (22).

The segmentation criterion offers additional healthcare cost-reducing incentives to members. But noticeably, most members (97.8%) do not qualify for the top categories—Diamond, Gold, and Silver. This percentage means that the majority of members are not entitled to the healthcare cost-reducing scheme. For members to forgo upgrading their category may reflect their economic landscape, as members may not have the capacity to purchase the minimum stock share for the top three categories. MFI needs to prioritise capacity-building to promote health and finances of its members (12).

The cooperative's health program has undergone changes in the past 5 years. Most of the health initiatives operate through collaborative partnerships, where both BMPC and its partners emphasised the health needs of their members and their capacities to deliver effective health services. Partnership agreements providing discount fees may contribute to improving health outcomes (23) of BMPC members.

To summarise, the design of the BMPC integrated health program uses the following structures.

### Subsidy or Outreach

Health initiatives under the subsidised mechanism include primary health care, feeding programs, I-Coop, blood donation and a dengue campaign. Except for the I-Coop where PhilHealth annual premiums are paid exclusively by BMPC, other health services function with partners such as PNRC, PhilHealth, a local primary school, and local medical facilities. The partners had already identified the health issues confronting the population in the locality.

The health services offered in this mechanism directly favour a reduction in healthcare costs for BMPC members. Beneficiaries of the PhilHealth annual premiums are assured that at least 30% of their hospitalisation bills and some outpatient charges are covered. Recipients of subscription glasses, dengue kits, and blood components are assured of cost-free health products. The feeding program may help reduce the burden of diseases caused by undernourishment and improve the health of children. Recently, BMPC-COVID 19 Community Assistance was established to provide food packs, personal protection equipment, and medical assistance (24).

The purpose of a medical mission is to physically bring health services and products closer to a group of the population. In doing so, BMPC members can take advantage of both the direct and indirect costs of healthcare services.

### Cooperative-Based Micro-Health Insurance and Health Loans

BMPC health microinsurance is structured using a cooperative-based health program wherein its provisions are governed in partnership with an HMC, 1CoopHealth. An HMC is deemed to be an alternative model for UHC coverage in the Philippines (25). Although BMPC and 1CoopHealth work together, each cooperative maintains autonomy in budgeting, staffing, and decision-making. The HMC focuses on a range of prioritised health services which helps reduce health service costs and eliminate unnecessary expenditures, thus making the programs sustainable (26). With an annual premium of US$75, clients get substantial financial health protection of at least US$1,200.

Acceptance of this health product from BMPC and its partner HMC is extremely low at 0.45%, similar to low enrolment of micro-health insurance in existing literature (27, 28). One factor for such low acceptance could be the misconception that HMO memberships are only for the rich (29). Additionally, since many cooperative members are poor, the low micro-health acceptance may also be caused by expenditure shocks from using other financial services (30). Another reason may be difficulty in understanding the health insurance policy (31).

In contrast to micro-health insurance, there is a higher acceptance of medical loans, though this could still be considered low at 6.1% from 2016 to 2018. Although low, BMPC records indicated that cooperative members find medical loan products more appealing than 1CoopHealth. There is no information to determine the reasons for the low uptake of medical loans among BMPC members. Microcredit can mitigate health shocks (13); however, the possibilities of using an alternative type of loan for health expenses (32) or selling assets to cover up health catastrophes (33) may be considered.

Medical loans are designed for large health expenses with lower interest rates and long repayment periods (34), or flexible repayment terms (35). But for the poor, healthcare expenditure is already catastrophic, and payments due for medical loans may mitigate healthcare costs, but not necessarily reduce them.

### Affordable Medicines and Patronage Refund

The pharmacy is both a cooperative business product and an extension health initiative to make pharmaceutical products affordable. Its net surplus operations indicated a high use of medicines and other pharmaceutical products among BMPC members. From a business perspective, its triple increase in sales volume indicates a positive business outcome. But with low take-ups of micro-health insurance and medical loans, the surge in pharmaceutical sales might need to be evaluated. No information was gathered to determine the grounds for this upsurge; however, it may indicate self-treatment practices (36) which are a popular approach for some common illnesses (37, 38), and were seen to be the first line of defence against illness in Bolivia, Burkina Faso, and Benin (14). Self-medication could create health risks that will increase the burden of disease and healthcare expenditure.

Cooperative ownership of the BMPC pharmacy provides equity shares to its members who enjoy patronage refunds each time they spend at the cooperative pharmacy. So aside from the advantage of purchasing lower-cost medications, cooperative members expect to receive dividends. The operation of the cooperative pharmacy indicates BMPC's attempt to apply profit generation into its business while providing medical care cost-reducing interventions to its members.

## Summary

Findings show that the cooperative's health program is designed to meet the needs of its members and partner organisations. Two significant factors anchored its health program design—capacities of the cooperative and its partners and the needs and capabilities of its target beneficiaries. This finding aligns with Ruducha's concept that the sustainability of an MFI health program is dependent on a client's health needs and its operational and financial capabilities.

The integrated microfinance-health program operate in three main structures—subsidised or outreach, microinsurance and health loans, and patronage refunds. In outreach and subsidised health initiatives, the cooperative engages with partners to deliver its activities. The cooperative gains the benefits of pooling resources and sharing of skills and expertise (39). Partner organisations like PhilHealth, PNRC and local primary schools have focused mandates. Health services rendered are to targeted populations, which helps the cooperative conserve financial resources. This finding supports Wright et al.'s (40) statement regarding the significance of health programs to set priorities such as the health needs of those people needing them most. It also supports Taylor and Marandi's (26) conclusion that prioritisation of healthcare needs will ensure MFI's health services efficiently use their resources to promote and support health among their members.

Other initiatives of the health program are business investments. Health initiatives anchored by microcredit business perspectives such as health loans and microinsurance aim to protect their members against OOP health expenditures, healthcare shocks and promote reductions in healthcare costs; however, they necessitate outlays which poor people would less likely prioritise. Despite its cost-reducing benefits and protection against OOP health expenditures, members are not inclined to enrol in these schemes. This finding is consistent with existing literature where microinsurance and medical loan uptakes were also low.

The cooperative's pharmaceutical business venture facilitates access to affordable medicine and affords financial viability to its members. With Generic Law and BMPC's reduced price, medicines are affordable. Access to quality affordable health products is embodied in UHC and is a significant feature of Ruducha's program design. Meanwhile, the health initiative's patronage refund set-up allows cooperative members to gradually build up their income.

There is no data available to help assess the output of completed health projects. Insufficient data was a significant gap that constrained the review of the program implementation. It is, thus, recommended for the program to create an improved mechanism for data collection.

Integrated microfinance-health programs could potentially facilitate reduction of healthcare costs, improve utilisation of health-related services among its members; thus, assist in achieving the objectives of *Healthy Philippines 2022* and UHC. This study confronted limitations. More studies that will evaluate the integrated MFI health initiatives are recommended to further identify gaps, outcome, or impacts of the program.

## Author Contributions

LA contributed to the concept, study design, review of literature, data collection and analysis, drafts, revisions, and preparation of the final draft. RK contributed to the design of the study, supervision of data analysis, and review of the manuscript. MR contributed to the data analysis and revisions of the manuscript. SN contributed to the research design and review of the article. All authors contributed to the article and approved the submitted version.

## Conflict of Interest

The authors declare that the research was conducted in the absence of any commercial or financial relationships that could be construed as a potential conflict of interest.
