# Supplementary material for: Combining Microfinance and Health in Reducing Poverty-Driven Healthcare Costs: Evidence From the Philippines
Source: Front Public Health. 2020 Oct 8;8:583455. doi: 10.3389/fpubh.2020.583455 (PMC7578378; doi:10.3389/fpubh.2020.583455)
Supplement: Supplementary file 1 [file Data_Sheet_1.PDF]

Supplemental data extracted from the BMPC Annual Reports

(Request granted to copy the provisions in following figures and table table from the BMPC annual reports)

Total number of BMPC members from 2013 – 2018[1]

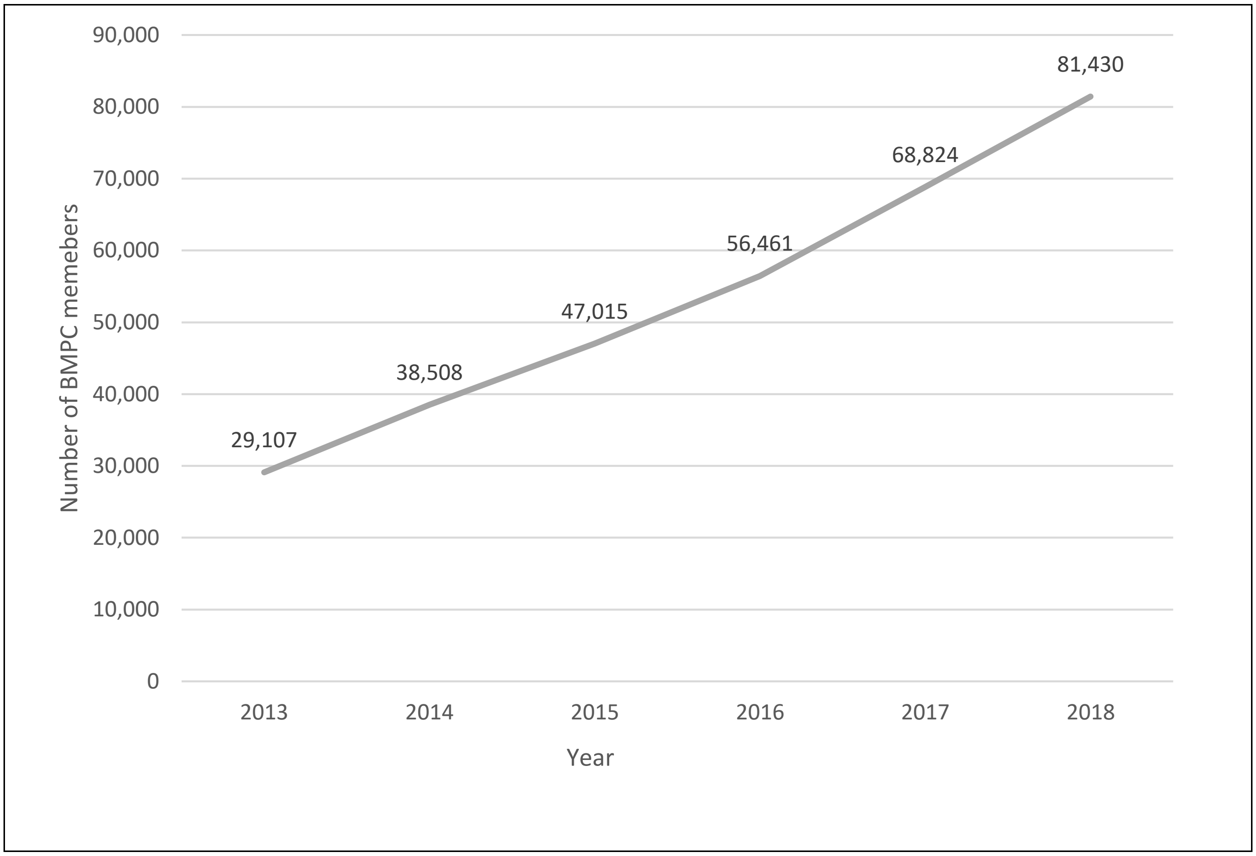

[1] Source of data: BMPC 2018 Annual Report

BMPC percentage distribution of members by branch

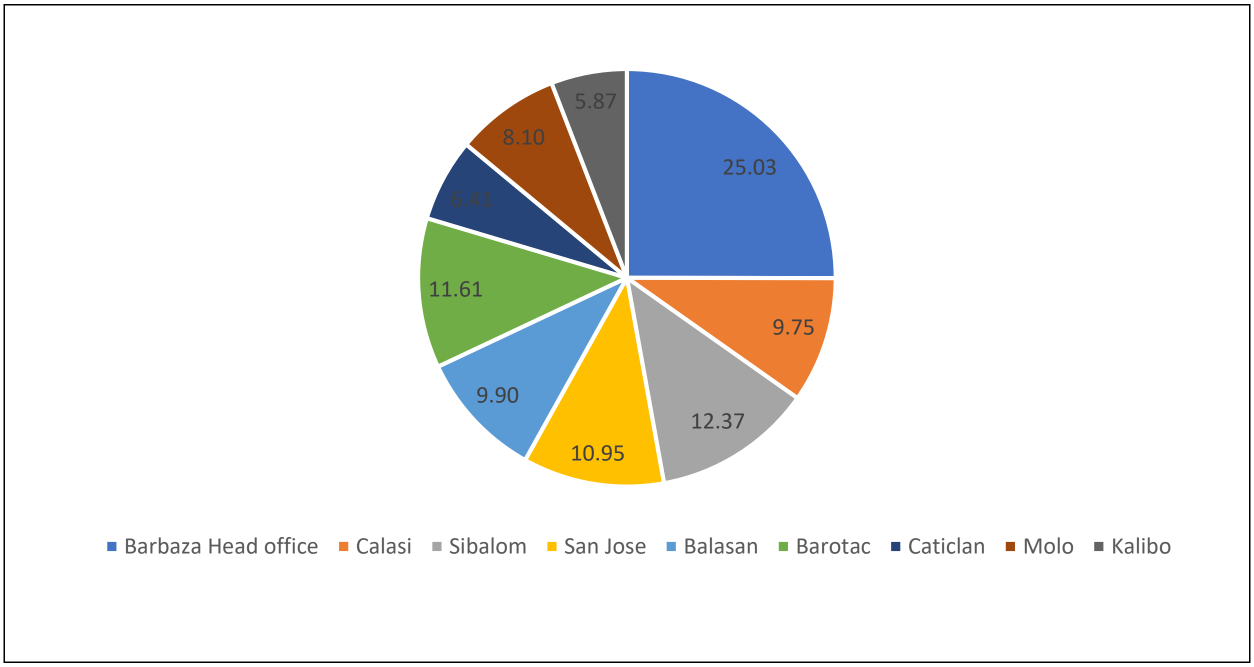

BMPC medical fund allocation in 2015 – 2018

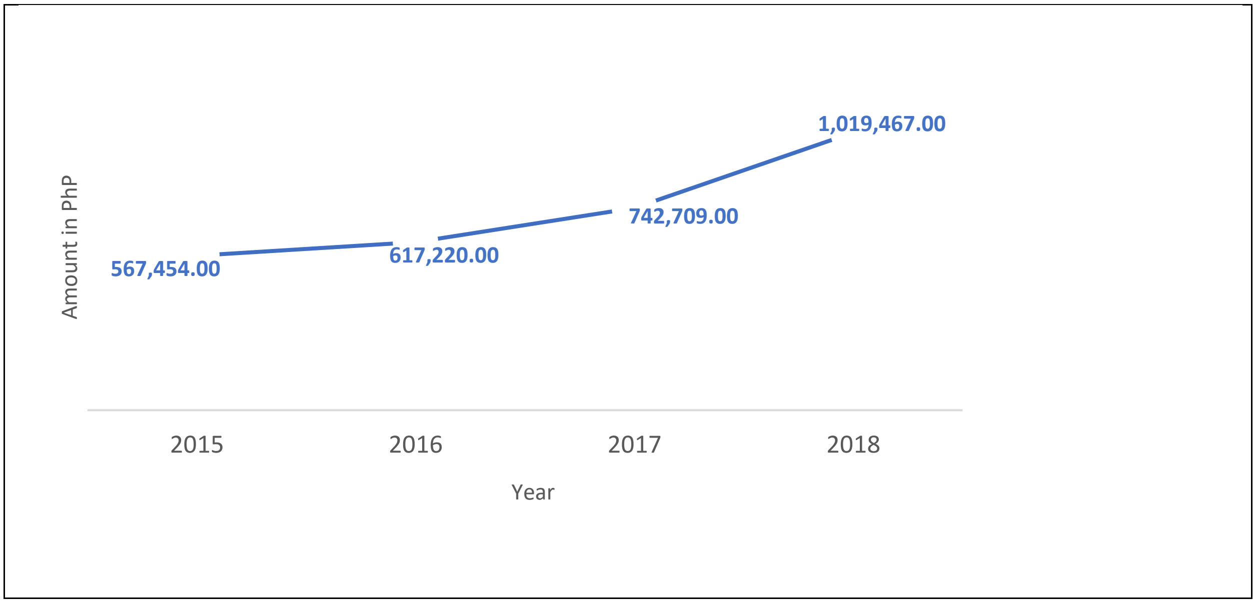

Percentage of BMPC members per branch who took up a medical loan from 2016 – 2018

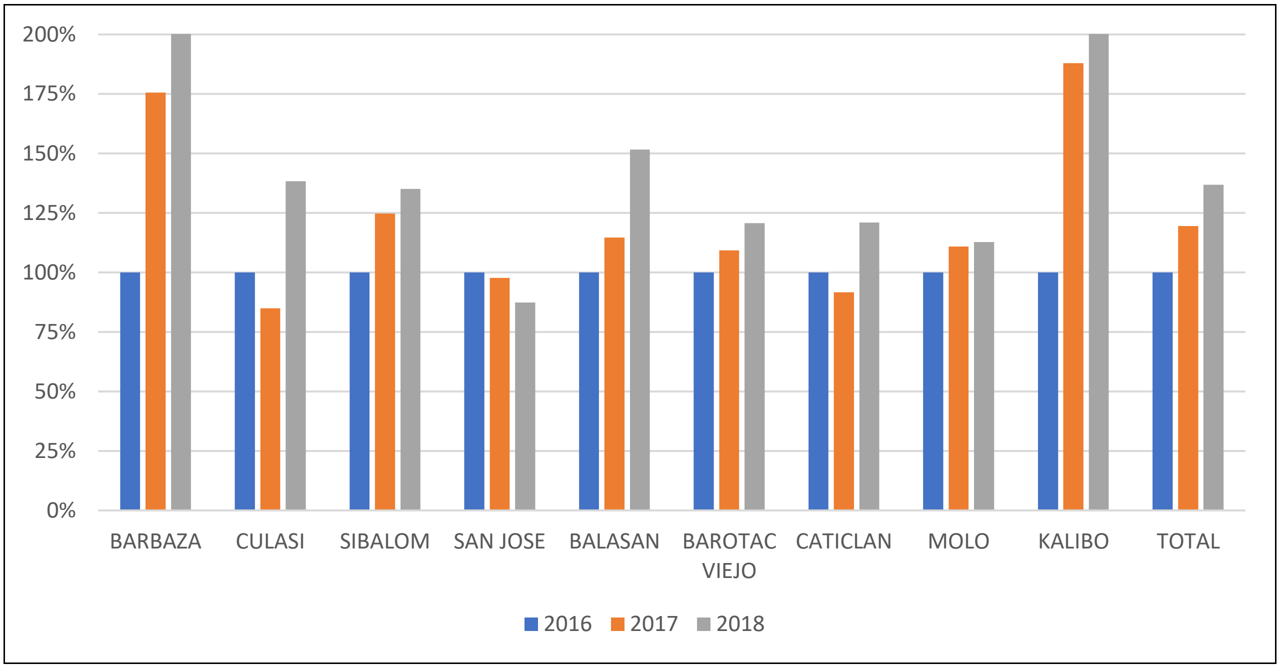

Total number of BMPC members and medical loan borrowers in 2016-2018

	2016	2017	2018
Number of borrowers	3504 *(6.2%)*	4188 *(6.1%)*	4793 *(5.9%)*
Total number of members	**56,461**	**68,824**	**81,430**

Number and percentage of BMPC members per category by branch in 2019

Branch	Segmentation Criteria								
	Category A				Category B				
	Silver		Gold		Diamond		Brass and Bronze		Total number of members in each branch
BARBAZA MAIN OFFICE	143	*0.70%*	248	*1.22%*	187	*0.92%*	19802	*97.16%*	20380
CULASI	58	*0.73%*	121	*1.52%*	46	*0.58%*	7715	*97.17%*	7940
SIBALOM	43	*0.43%*	71	*0.70%*	33	*0.33%*	9928	*98.54%*	10075
SAN JOSE	82	*0.92%*	81	*0.91%*	50	*0.56%*	8705	*97.61%*	8918
BALASAN	68	*0.85%*	89	*1.11%*	58	*0.72%*	7801	*97.32%*	8016
BAROTAC VIEJO	94	*0.99%*	56	*0.59%*	17	*0.18%*	9287	*98.23%*	9454
CATICLAN	50	*0.96%*	76	*1.46%*	21	*0.40%*	5076	*97.19%*	5223
MOLO	68	*1.03%*	61	*0.92%*	0	*0.00%*	6466	*98.04%*	6595
KALIBO	99	*2.07%*	0	*0.00%*	0	*0.00%*	4685	*97.93%*	4784
**Total**	**705**	***0.84%***	**803**	***0.95%***	**412**	***0.49%***	**82510**	***97.73%***	**84430**
