# Supplementary material for: Combining Microfinance and Health in Reducing Poverty-Driven Healthcare Costs: Evidence From the Philippines
Source: Front Public Health. 2020 Oct 8;8:583455. doi: 10.3389/fpubh.2020.583455 (PMC7578378; doi:10.3389/fpubh.2020.583455)
Supplement: Supplementary file 2 [file Data_Sheet_2.PDF]

Appendages:

Appendix A: List of review documents

CDA Memorandum Circular 2018-01 Revised Guidelines on Social Audit of Cooperatives

BMPC reports

- Membership report 2016 - 2018
- Descriptive report of annual bloodletting
- Medical Fund allotment 2015 – 2018
- Cooperative blood bank fund 2017 – 2018
- Cooperative Assurance Centre report 2016 – 2018
- Social Services report 2016 – 2018
- Cooperative pharmacy revenue report 2017 – 2018
- Medical loan report 2016 – 2018
- Annual plan report (Goal 7: To enhance and strengthen coop's advocacy programs.) 2017-2018

Cooperative Health Management Federation 2014 Annual report

I Cooperative Insurance System of the Philippines 2014 - 2017 Annual Report

Appendix B: Segmentation criteria re-classification

(Request granted to copy the provisions in this table from the 2018 BMPC annual report)	
**CATEGORY A**	**CATEGORY B**
**Diamond**	**Bronze**
At least five (5) years as a regular member.	At least two (2) years as a regular member.
Share capital of at least P15,000.00 with additional deposit of at least P500.00 annually for the last two (2) years.	Share capital of at least P3,000.00 with additional deposit of at least P150.00 annually for the last two (2) years.
Attended the District Assembly at least once for the last two (2) years.	Attended the District Assembly at least once for the last two (2) years
Attended at least 50% of the Ownership/ MES in a year for the last two (2) years.	Regular monthly savings deposit of at least P10.00 or P30.00 in a quarter for the last two (2) years.
Regular monthly savings deposit of at least P50.00 or P150.00 in a quarter for the last two (2) years.<br>No loan amortisation in default for the last two (2) years.<br>**Gold**<br>At least four (4) years as a regular member.<br>Share capital of at least P10,000.00 with additional deposit of at P350.00 annually for the last two (2) years.<br>Attended the District Assembly at least once for the last two (2) years.<br>Attended at least 50% of the Ownership/ MES in a year for the last two (2) years.<br>Regular monthly savings deposit of at least P90.00 in a quarter for the last two (2) years.<br>No loan amortisation in default for the last two (2) years.<br>**Silver**<br>At least three (3) years as a regular member.<br>Share capital of at least P5,000.00 with additional deposit of at least P200.00 annually for the last two (2) years<br>Attended the District Assembly at least once for the last two (2) years.<br>Attended at least 50% of the Ownership/ MES in a year for the last two (2) years.<br>Regular monthly savings deposit of at least P20.00 or P60.00 in a quarter for the last two (2) years.<br>No loan amortisation in default for more than 90 days for the last two (2) years.<br>No loan amortisation in default for more than 90 days for the last two (2) years.	No loan amortisation in default for more than 30 days for the last two (2) years.<br>**Brass**<br>At least two (2) years as a regular member.<br>Share capital of at least P3,000.00 with additional monthly deposit of at least P10.00

Appendix C: Number and percentage of BMPC members per category by branch

(Request granted to copy the provisions in this table from the 2018 BMPC annual report)					
Branch/Category	Silver	Gold	Diamond	Brass and Bronze	Total number of members in each branch
BARBAZA MAIN OFFICE	143	248	187	19802	20380
CULASI	58	121	46	7715	7940
SIBALOM	43	71	33	9928	10075
SAN JOSE	82	81	50	8705	8918
BALASAN	68	89	58	7801	8016
BAROTAC VIEJO	94	56	17	9287	9454
CATICLAN	50	76	21	5076	5223
MOLO	68	61		6466	6595
KALIBO	99			4685	4784
Total	705	803	412	82510	84430
**Percentage**	**0.84%**	**0.95%**	**0.49%**	**97.73%**	**100%**

Appendix D: Hospitalisation benefits for BMPC members based on segmentation criteria

(Request granted to copy the provisions in this table from the 2018 BMPC annual report)				
Member Category	Hospitalisation benefit per day	Minimum days confinement	Maximum days confinement	Availed confinement per year
Diamond	PhP 500	3	5 days	1
Gold	PhP 400	3	5 days	1
Silver	PhP 300	3	5 days	1
Bronze	0	0	0	0
Brass	0	0	0	0
